# Structure and proteomic analysis of the crown-of-thorns starfish (*Acanthaster sp.*) radial nerve cord

**DOI:** 10.1038/s41598-023-30425-1

**Published:** 2023-02-27

**Authors:** Meaghan K. Smith, Bronwyn A. Rotgans, Tomas Lang, Ryan Johnston, Tianfang Wang, Saowaros Suwansa-ard, Utpal Bose, Nori Satoh, Michaela Egertova, Michael R. Hall, Maria Byrne, Maurice R. Elphick, Cherie A. Motti, Scott F. Cummins

**Affiliations:** 1 Centre for Bioinnovation, University of the Sunshine Coast, Maroochydore DC, QLD 4558 Australia; 2 School of Science, Technology and Engineering, University of the Sunshine Coast, Maroochydore DC, QLD 4558 Australia; 3 Okinawa Institute of Science and Technology, Okinawa, Japan; 4 School of Biological and Chemical Sciences, Queen Mary University of London, London, UK; 5 Australian Institute of Marine Science (AIMS), Cape Ferguson, Townsville, QLD 4810 Australia; 6 School of Life and Environmental Sciences, University of Sydney, Sydney, NSW 2006 Australia

**Keywords:** Gene expression analysis, Proteomic analysis, Molecular neuroscience, Neurophysiology

## Abstract

The nervous system of the Asteroidea (starfish or seastar) consists of radial nerve cords (RNCs) that interconnect with a ring nerve. Despite its relative simplicity, it facilitates the movement of multiple arms and numerous tube feet, as well as regeneration of damaged limbs. Here, we investigated the RNC ultrastructure and its molecular components within the of Pacific crown-of-thorns starfish (COTS; *Acanthaster sp*.), a well-known coral predator that in high-density outbreaks has major ecological impacts on coral reefs. We describe the presence of an array of unique small bulbous bulbs (40–100 μm diameter) that project from the ectoneural region of the adult RNC. Each comprise large secretory-like cells and prominent cilia. In contrast, juvenile COTS and its congener *Acanthaster brevispinus* lack these features, both of which are non-corallivorous. Proteomic analysis of the RNC (and isolated neural bulbs) provides the first comprehensive echinoderm protein database for neural tissue, including numerous secreted proteins associated with signalling, transport and defence. The neural bulbs contained several neuropeptides (e.g., bombyxin-type, starfish myorelaxant peptide, secretogranin 7B2-like, Ap15a-like, and ApNp35) and Deleted in Malignant Brain Tumor 1-like proteins. In summary, this study provides a new insight into the novel traits of COTS, a major pest on coral reefs, and a proteomics resource that can be used to develop (bio)control strategies and understand molecular mechanisms of regeneration.

## Introduction

Although seastars and other echinoderms are not cephalised, they have a well-developed nervous system, which includes a circumoral nerve ring and radial nerve cords (RNCs)^1^. Due to their position as basal deuterostomes, there has been much interest in the echinoderm nervous system with respect to the evolution of the neural system of vertebrates^2^. Similar to sea urchins and brittle stars, each seastar’s RNC comprises of an inner hypo- and outer ectoneural system^3^, whereby the ectoneural system has a distinct neuroepithelium that forms with the surface ectoderm in the region of the RNC. This neuroectoderm presents a smooth external surface and is comprised of neuronal cell bodies and an underlying neuropile that contains axons and supporting neuroglia, while the inner hyponeural system contains motoneurons^3,4^.

The multi-armed crown-of-thorns starfish (COTS; *Acanthaster sp*, formerly called *Acanthaster planci*) are unusual among seastars, being specialised coral predators and large in size. As adults, an individual COTS has up to 22 arms and is covered with toxic protective spines^5^, can consume up to 10 m^2^ of coral tissue annually^6^. Outbreaks of COTS cause massive coral loss and, in some regions of the Great Barrier Reef, are thought to be responsible for up to 42% of coral death^7–10^. While COTS have been well studied with respect to population outbreaks^11^, toxins^12^, life cycle^13^ and vision^14–16^, little is known about other aspects of their biology, including neurobiology. As well, their ability to rapidly regenerate damaged limbs, a major contributor to their survival, has had limited investigation, besides some morphological experiments^17^.

To obtain insights into the molecular neurobiology of COTS, our previous studies have identified neuropeptides and small molecule neurotransmitters present in the RNC^18,19^. Here, we further analysed the anatomy and ultrastructure of the RNC, revealing an array of small bulb-like structures (hence called neural bulbs) that extend from the ectoneural surface, and may represent unique adaptations to corallivory. In addition, we established a comprehensive RNC proteomic dataset, including putative secreted proteins. This protein resource provides a foundation for the investigation of control strategies, as well as to understand fundamental processes such as arm regeneration at the molecular level.

## Methods

### Tissue collection

**Figure 1:**
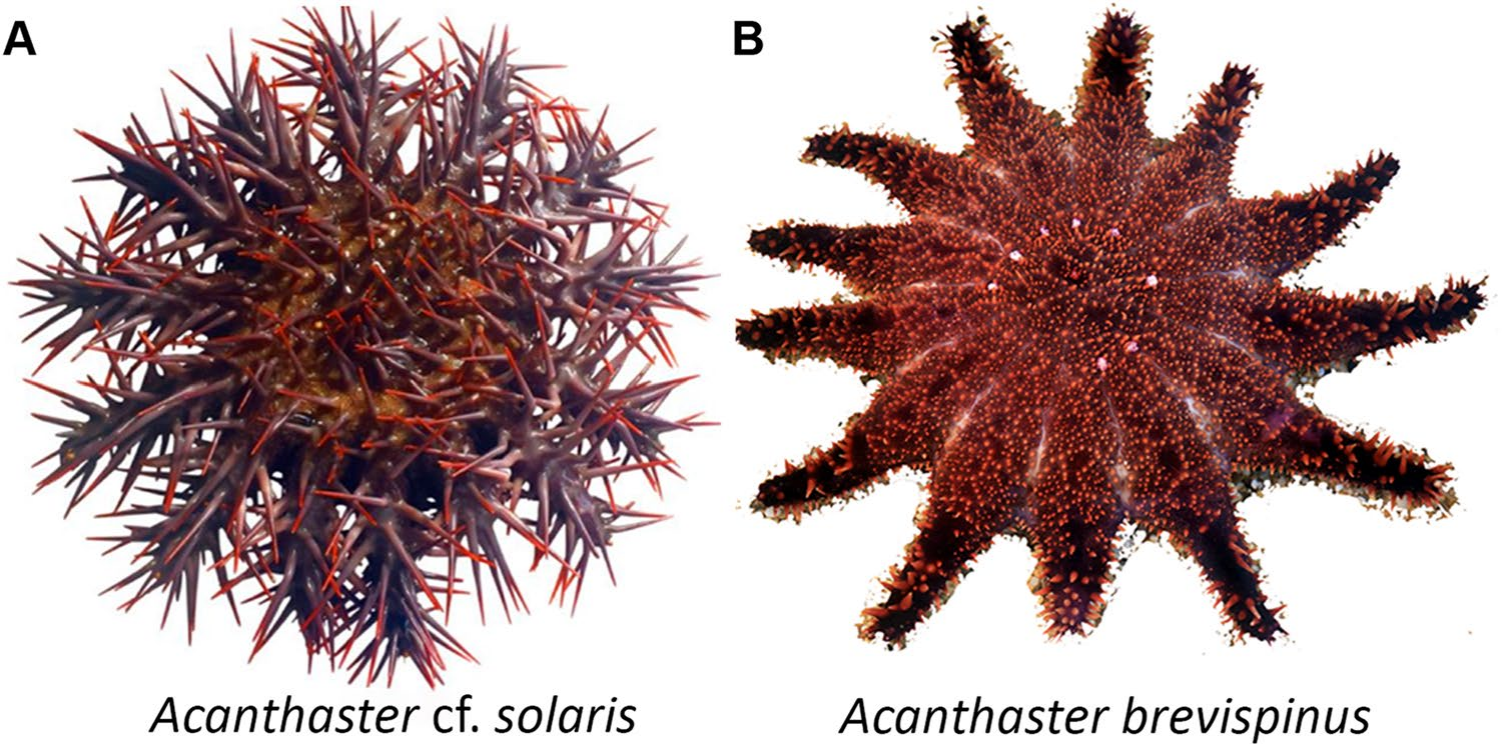
Images of crown-of-thorns species. (**A**) Crown-of-thorns starfish, *Acanthaster* sp., and (**B**) Short-spined crown-of-thorns starfish, *Acanthaster brevispinus*.

Adult (> 30 cm) and juvenile COTS (< 2 cm) (Fig. 1A) were collected by the Association of Marine Park Tourism Operators Pty Ltd (AMPTO) from the Great Barrier Reef in Far North Queensland, Australia (permit G17/38,293.1). Adults of unknown sex were housed at Sea Life Aquarium, Mooloolaba, for 1–2 weeks before arms were dissected to isolate the ambulacrum (which contains the RNC) or the RNC only. An adult of *A. brevispinus* (Fig. 1B), also of unknown sex, was collected by trawler off Townsville, Australia (Permit G17/38,293.1), housed at the Australian Institute of Marine Science and was similarly dissected. Whole animal images of COTS and *A. brevispinus* were taken using an Apple iPhone 6.

### Histological examination and microscopy

The RNC tissues were processed for light microscopy and scanning (SEM) and transmission (TEM) electron microscopy. For histology, the tissue was fixed in 4% paraformaldehyde overnight at 4 °C, then subjected to three 15 min-washing cycles in 70% ethanol/1 × phosphate buffered saline (PBS). Subsequently, tissues were placed into Morse’s solution (20% formic acid, 10% sodium citrate mixture) for 2 h in order to decalcify ossicles before being processed for a routine wax embedding. The sections (10-µm thick) were stained using haematoxylin and eosin (H&E) and/or trichrome methods^20^, visualised under a compound light microscope, and then photographed. For SEM and TEM, RNCs were fixed in a mixture of 2.5% glutaraldehyde and 2% paraformaldehyde in 0.1 M Millonig’s buffer for 4 h at room temperature, followed by three 10-min washes in this same buffer. They were then fixed in 4% osmium tetroxide in 0.1 M Millonig’s buffer for 1 h and again washed in this buffer and rinsed with MilliQ water. The tissues were dehydrated through a graded ethanol series up to 100%. For SEM, the specimens were critical point dried, and mounted onto stubs with carbon tape for thin layer gold sputter coating (Hitachi E5000) for 1 min. The specimens were examined using a Hitachi S-2500 SEM at 15 kV. For TEM, the tissue was block stained using saturated uranyl acetate, processed for embedding in Araldite 502 and sectioned (90-nm thick) using a diamond knife. Sections were mounted on slot grids coated with formvar. Images were taken at a range of magnifications on a JEOL transmission electron microscope at 4-megapixel resolution.

### Proteomic analysis of the adult COTS radial nerve cord and neural bulbs

For proteomic analysis, RNC tissues from adult COTS were carefully collected and immediately snap frozen in liquid nitrogen and stored at − 80 °C for protein isolation. An overall workflow for the proteomic analysis of the adult COTS RNC is summarised in Fig. S1. In brief, adult RNC tissue (~ 200 mg) was homogenised in 0.1% trifluoroacetic acid (TFA). The homogenate was then centrifuged at 12,000×*g* for 15 min. The supernatant (i.e., soluble proteins) and pellet (i.e., insoluble proteins) were collected and processed through reverse-phase high pressure liquid chromatography (RP-HPLC) and protein gel electrophoresis (SDS-PAGE), respectively.

Prior to RP-HPLC, the supernatant was loaded onto C_18_ Sep-Pak cartridges (Waters) following the manufacturer’s protocol, and proteins eluted with 60% acetonitrile (CH_3_CN). The eluate was lyophilised using a Savant vacuum dryer before being resuspended in 0.1% TFA. Fractionation by RP-HPLC was achieved on a semi-preparative Agilent-bio C_18_ HPLC (1 × 15 cm) column using a one-step linear gradient (0–60% CH_3_CN containing 0.1% TFA for 60 min, with a flow rate of 0.5 ml/min). Fractions were collected at 5-min intervals, lyophilised, then reconstituted in 200 µl of MilliQ water for in-solution trypsin digestion, performed as described previously^18^.

The RNC pellet was rehydrated with SDS-PAGE buffer solution (Bio-Rad) containing 200 mM dithiothreitol and heated for 10 min at 56 °C. Samples were loaded onto a Bio-Rad Mini-Protean TGX precast 4–20% SDS-PAGE gel (Bio-Rad) and run at 110 V for 1 h. The gel was transferred into an isopropanol fixing solution for 60 min at room temperature, then immersed in Coomassie Blue stain (Bio-Rad) for 2 h at room temperature with rocking. Finally, the stain was removed, and gel was washed twice in 10% acetic acid with rocking for 30 min each. Protein bands were excised with a sterile scalpel, followed by in-gel trypsin digestion using a standard protocol^21^.

For neural bulb protein identification, separated adult COTS RNCs were immediately placed in a petri dish on ice with sufficient filtered artificial seawater to just cover the tissue (~ 1 ml). Under a dissecting microscope, neural bulbs were carefully removed using a scalpel blade and immersed in 100 μl of artificial seawater. Following removal, the remaining radial nerve was observed under a compound microscope (Leica DM5500). The neural bulb sample was acidified to 0.1% TFA, then homogenised and centrifuged at 12,000×*g* for 15 min. Supernatant was collected and purified through C_18_ Sep-Pak cartridges. Proteins were eluted with 70% CH_3_CN containing 0.5% TFA. Eluted samples were lyophilised before being subjected to in-solution trypsin digestion^18^.

Tryptic peptides were prepared in 10 μl 0.5% aqueous formic acid (FA) and analysed by liquid chromatography with tandem mass spectrometry (LC–MS/MS) on an ExionLC liquid chromatography system (AB SCIEX, Concord, Canada) coupled to a quadrupole time-of-flight (QTOF) X500R mass spectrometer (AB SCIEX, Concord, Canada) equipped with an electrospray ion source. Ten microlitres of each sample was injected onto a 100 mm × 2.1 μm Aeris PEPTIDE XB-C18 100 uHPLC column (Phenomenex, Sydney, Australia) equipped with a Security Guard column for MS/MS analysis. Two mobile phases were used: solvent A [0.1% FA (aq)] and solvent B [100% CH_3_CN/0.1% FA (aq)]. Stepwise linear gradients of 5–35% solvent B over 10 min at 400 µl/min flow rate, followed by a steeper gradient from 35 to 80% solvent B in 2 min and 80–95% solvent B in 1 min were used for peptide elution. Solvent B was held at 95% for 1 min to wash the column and returned to 5% solvent B for equilibration prior to the next sample injection. The ion spray voltage was set to 5500 V, de-clustering potential (DP) 100 V, curtain gas flow 30, ion source gas (1) 40, ion source gas (2) 50 and spray temperature at 450 °C. Mass spectral data was acquired in an Information Dependant Acquisition mode. Full scan QTOF-MS data was acquired over the mass range 350–1400 and for product ion MS/MS 50–1800. Ions observed in the QTOF–MS scan exceeding a threshold of 100 cps and a charge state of + 2 to + 5 were set to trigger the acquisition of product ion. The data was acquired and processed using PEAKS studio (Bioinformatics Solutions Inc., Waterloo, ON, Canada, version 7.0) with the assistance of MS Data Converter (Beta 1.3; https://sciex.com). Peptides were analysed using PEAKS v7.0 (BSI, Canada) against the protein database assembled from the available COTS transcriptomes and genome data (http://marinegenomics.oist.jp)^22^.

### Protein annotations and secretome analysis

Identified proteins were subject to BLAST search via the National Centre for Biotechnology Information (NCBI) site (http://www.ncbi.nlm.nih.gov) using non-redundant protein sequences and nucleotide collection of NCBI. For secretome analysis, predicted secreted proteins were analysed based on two aspects, including the presence of a signal peptide at the N-terminus and the absence of a transmembrane domain within the coding region. N-terminal signal sequences and transmembrane protein domains were predicted using the SignalP (Ver. 5.0)^23^ and TMHMM (Ver. 2.0)^24^ programs, respectively. Protein functional annotations from the COTS genome database provided by Okinawa Institute of Science and Technology (https://marinegenomics.oist.jp), including the Gene Ontology (GO) annotations, were downloaded for further investigation. Based on the GO cellular component annotation, a representative subcellular localization for those identified proteins was assigned. Furthermore, all GO biological processes and molecular function annotations for all identified proteins were counted, and the top 10 functional groups for those identified proteins compiled and considered in the context of echinoderm biology. Based on transcriptome data on the COTS genome database (http://marinegenomics.oist.jp)^22^, expression of genes encoding all identified proteins was retrieved and used for heatmap production, showing relative tissue gene expression of each identified protein. Heatmaps were prepared using the ClustVis web tool (https://biit.cs.ut.ee/clustvis/)^25^. Protein diagram schematics were prepared using the Illustrator for Biological Sequences web tool (http://ibs.biocuckoo.org/userguide.php)^26^.

## Results

### Structure of the radial nerve cord in COTS and *A. brevispinus*

**Figure 2:**
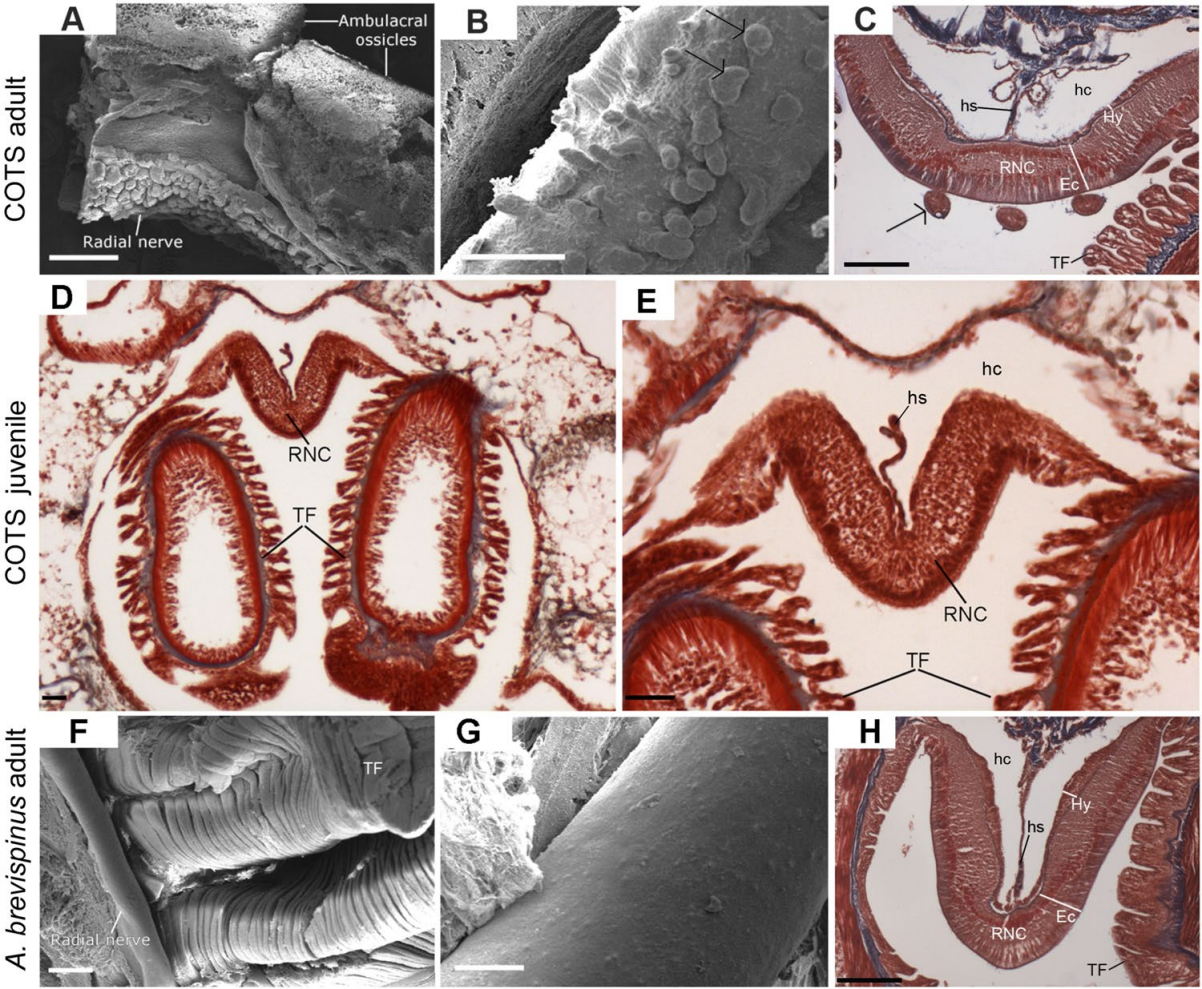
Scanning electron microscope (SEM) and histology (trichrome stain) of the radial nerve cord (RNC). (**A**–**C**) Adult COTS: (**A**) SEM of the RNC and adjacent ambulacral ossicles. The tissue bordering the neural bulbs is a cut away section of epithelium; (**B**) SEM of the RNC, showing neural bulbs (arrows); (**C**) Histology, showing neural bulbs (arrow) protruding from the ectoneural surface of the RNC. (**D**,**E**) Juvenile COTS: Histology, showing the smooth surface of the RNC and absence of the bulbous structures seen in adult. (**F**–**H**) *A. brevispinus*: (**F**,**G**) SEM of the RNC, showing its smooth ectoneural surface; (**H**) Histology, showing the absence of neural bulbs on the ectoneural surface of the RNC. In histology, blue represents connective tissue, dark red/purple indicates nuclei and red/pink is cytoplasm. *TF*, Tube foot; *Ec*, ectoneural layer; *Hy*, hyponeural layer; *hs*, haemal sinus; *hc*, hyponeural coelom. Scale bars (**A**,**F**) = 500 μm; (**B**,**G**) = 200 μm; (**C**,**H**) = 50 μm; (**D**,**E**) = 40 µm.

The general morphology of the seastar RNC has been described previously^27^. As in other seastars, the COTS RNC has a V-shaped appearance when viewed in transverse section (possibly an artefact of contraction) and extends along the oral surface between rows of locomotory tube feet. The RNC comprises an outer ectoneural system and an inner hyponeural system, while the haemal sinus was running along the “V” space of the hyponeural coelom. The radial water canal of the water vascular system and the hyponeural coelom run parallel to the RNC along the arm. Close examination of the adult COTS RNC surface using SEM and histology trichrome-stained transverse sections revealed that the ectoneural surface is covered with small (40–100 μm diameter) bulbous structures, the neural bulbs (Fig. 2A–C). In contrast, histological analysis revealed that juvenile COTS RNC have a smooth ectoneural surface (Fig. 2D,E). The RNC of adult COTS has neural bulbs in contrast with the smooth ectoneural surface of the RNC in *A. brevispinus*, also shown by SEM and histology (Fig. 2F–H).

**Figure 3:**
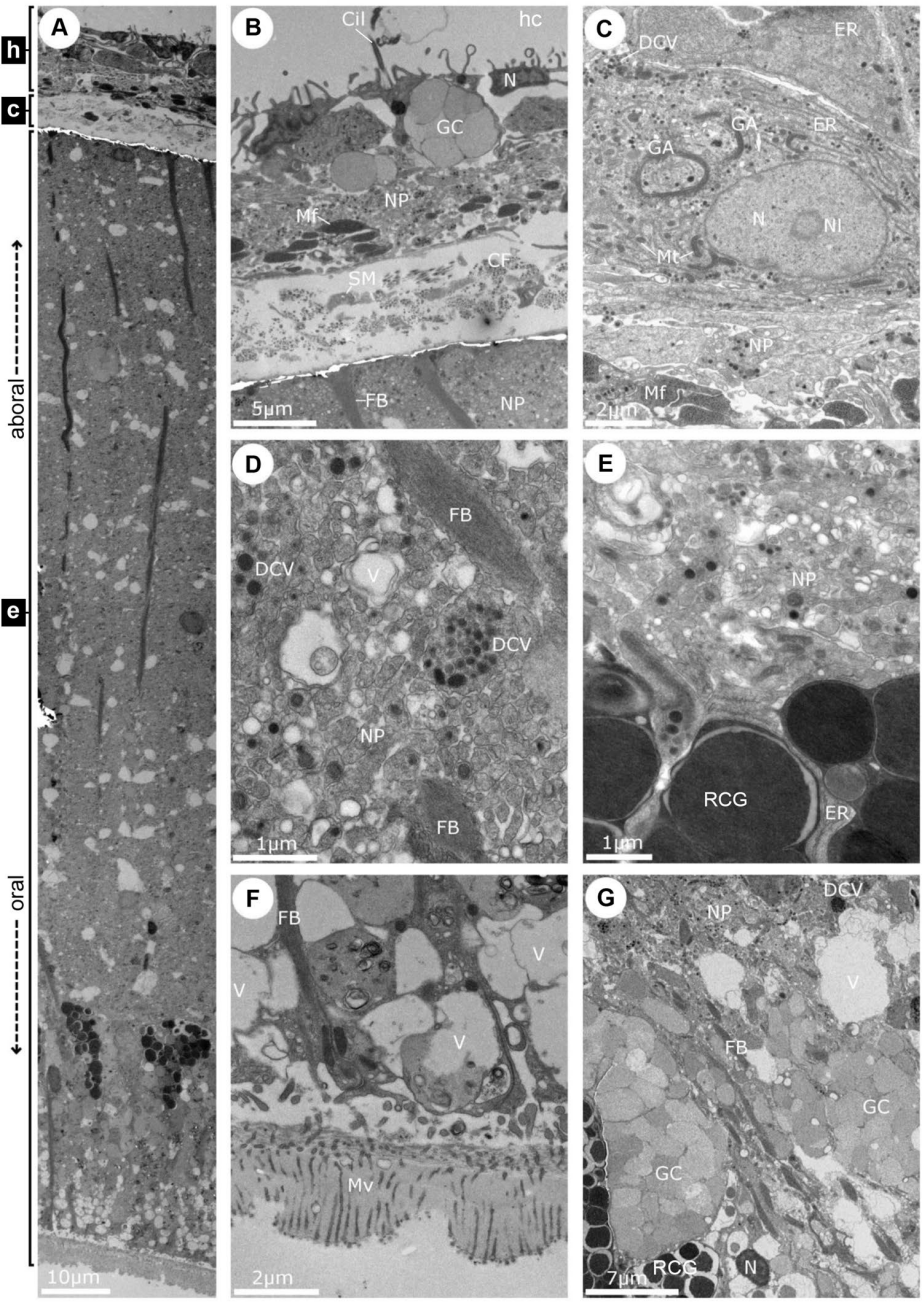
TEM of the adult COTS radial nerve cord (RNC). (**A**) Transverse section showing the hyponeural region (h), connective tissue layer (c), and ectoneural region (e). (**B**) Hyponeural region, connective tissue layer, and uppermost part of the ectoneural neuropile. (**C**) High-magnification view of a large cell in the hyponeural region with a portion of the neuropile and myofilaments visible. (**D**) Ectoneural neuropile showing, dense-cored vesicles and the fibre bundle of the radial-glia-like process. (**E**) Lower border of the ectoneural neuropile, with dense protein vesicle-like cell granules surrounded by endoplasmic reticulum. (**F**) Neuroepithelium containing large vesiculated cells and the extracellular cuticle-like cover through which microvilli of the epithelial cells traverse to the oral surface; this is the lowermost portion of the ectoneural tissue and is the surface exposed to the seawater environment. (**G**) Goblet-like cells from the epithelial portion of the ectoneural tissue. *CF* collagen fibres, *Cil* cilium, *DCV* dense-cored vesicles, *ER* endoplasmic reticulum, *FB* fibre bundles, *GA* Golgi apparatus, *GC* goblet-like cell, *hc* hyponeural coelom, *Mf* myofilament, *Mt* mitochondria, *Mv* microvilli, *N* nucleus, *Nl* nucleolus, *NP* neural plexus, *RCG* releasing-like cell granule, *SM* smooth muscle cell-like, *V* vacuole.

In TEM sections, the RNC of adult COTS is comprised of the thin inner hyponeural region and the outer thicker ectoneural region, and these were separated by a thin layer of connective tissue (Fig. 3A). A cross-section of the entire RNC showed its layers. The hyponeural region had small bundles of axons forming a neuropile and its upper-most region was covered by the epithelial lining of the hyponeural coelom (Fig. 3B). This epithelium also had squamous cells that gave rise to microvilli and cilia that extended into the coelom (Fig. 3A,B). Cell bodies in this region were often large and well-populated with protoplasmic structures and sporadic goblet-like cells filled with large vesicles. In the larger cells, the nucleoli could frequently be distinguished within relatively large nuclei; these were surrounded by elongated mitochondria, Golgi apparatus, endoplasmic reticulum, and many dark dense-cored granules (Fig. 3C,D). These dense-cored vesicles were distributed throughout the underlying neural plexus, as well as interposed with myofilaments that did not occur in the rest of the tissue (Fig. 3D). Inferior to the hyponeural plexus and separated by a narrow basement membrane was the connective tissue layer. It was comprised of longitudinally arranged collagen fibres, matrix and occasional cells (Fig. 3A,B).

The ectoneural region contained relatively enlarged nerve fibres with abundant synaptic vesicles and were covered by a prominent and thick extracellular layer of cuticle-like material through which microvilli of the neuroepithelial cells traversed (Fig. 3A,E–G). The most prominent structures included neuronal somas, goblet-like cells, fibre bundles, large granular secretory vesicles, the nuclei of tall thin epithelial cells that were associated with chains of vacuoles, and the abundant microvilli. Long fibre bundles traversed the ectoneural layer and may arise from glial-like cells.

**Figure 4:**
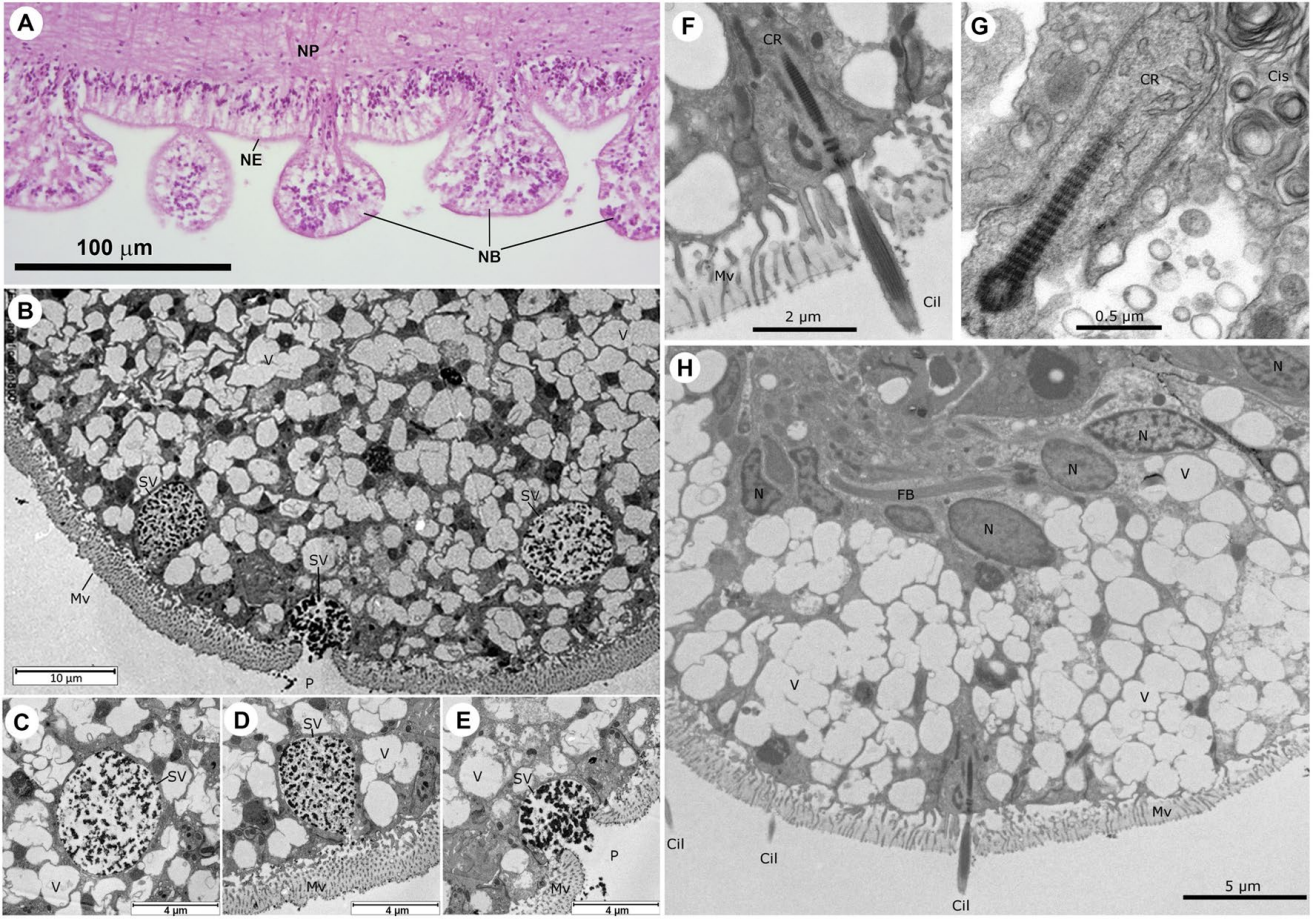
Histology and TEM of the neural bulbs of COTS radial nerve cord (RNC). (**A**) Histology of the ectoneural surface (H&E stain) (**B**) TEM of bulb edge showing what may be secretory vesicles, containing electron dense diffuse material at various stages of activity leading to exocytosis of contents into the external space. (**C**) Secretory vesicle located at ~ 10 μm from the microvilli border. (**D**) Secretory vesicle fusing with the outer surface of the bulb, note the liberated granular contents visible at the base of the microvilli layer. (**E**) Secretory vesicle fused with an adjacent microvilli layer, forming a small pore, and releasing its granular contents to the exterior environment. (**F**) Cilium with rootlet projecting through the microvilli of the epithelial surface. (**G**) High magnification view of ciliary rootlet and a small corner of the endoplasmic reticulum cisternae, from which light cored vesicles can be seen emerging. (**H**) Epithelial region of ectoneural tissue populated with multiple cilia projecting past the microvilli layer. *Cil* cilium, *Cis* cisternae of endoplasmic reticulum, *CR* ciliary rootlet, *FB* fibre bundles, *Mv* microvilli, *N* nucleus, *NB* neural bulb, *NE* neuroepithelium, *NP* neural plexus, *P* pore, *SV* secretory Vesicle, *V* vacuole.

Unique to adult COTS, the ectoneural epithelial cells give rise to bulbous extensions that appear to protrude to the external (outer) environment (Fig. 4A). Histologically, these neural bulbs appear to be extensions of the neuroepithelium with a similar composition. Examination of the neural bulbs with TEM, revealed that they are dominated by large secretory-like vesicles, the contents of which may have been lost in tissue processing (Fig. 4B). Amongst the empty appearing vesicles were larger vesicles with electron dense diffuse contents, potentially proteinaceous. The different sizes of these vesicles indicated that they were at various transitional stages of their activity and that the contents were destined for export to the exterior. Some of these vesicles were situated close to the microvilli border (Fig. 4C) or merging with it (Fig. 4D), forming a small pore to release its contents (Fig. 4E). It is possible, however not apparent in the images, that glial-like cells connect with the long fibre bundles, giving rise to cilia that project beyond the microvilli border (Fig. 4F–H). A section of the adjacent ectoneural surface revealed a similar appearance with an abundance of empty appearing vesicles, but the vesicles with the electron dense inclusions were not present in this area (Fig. 4H).

### Proteomic analysis of the adult COTS radial nerve cord

**Figure 5:**
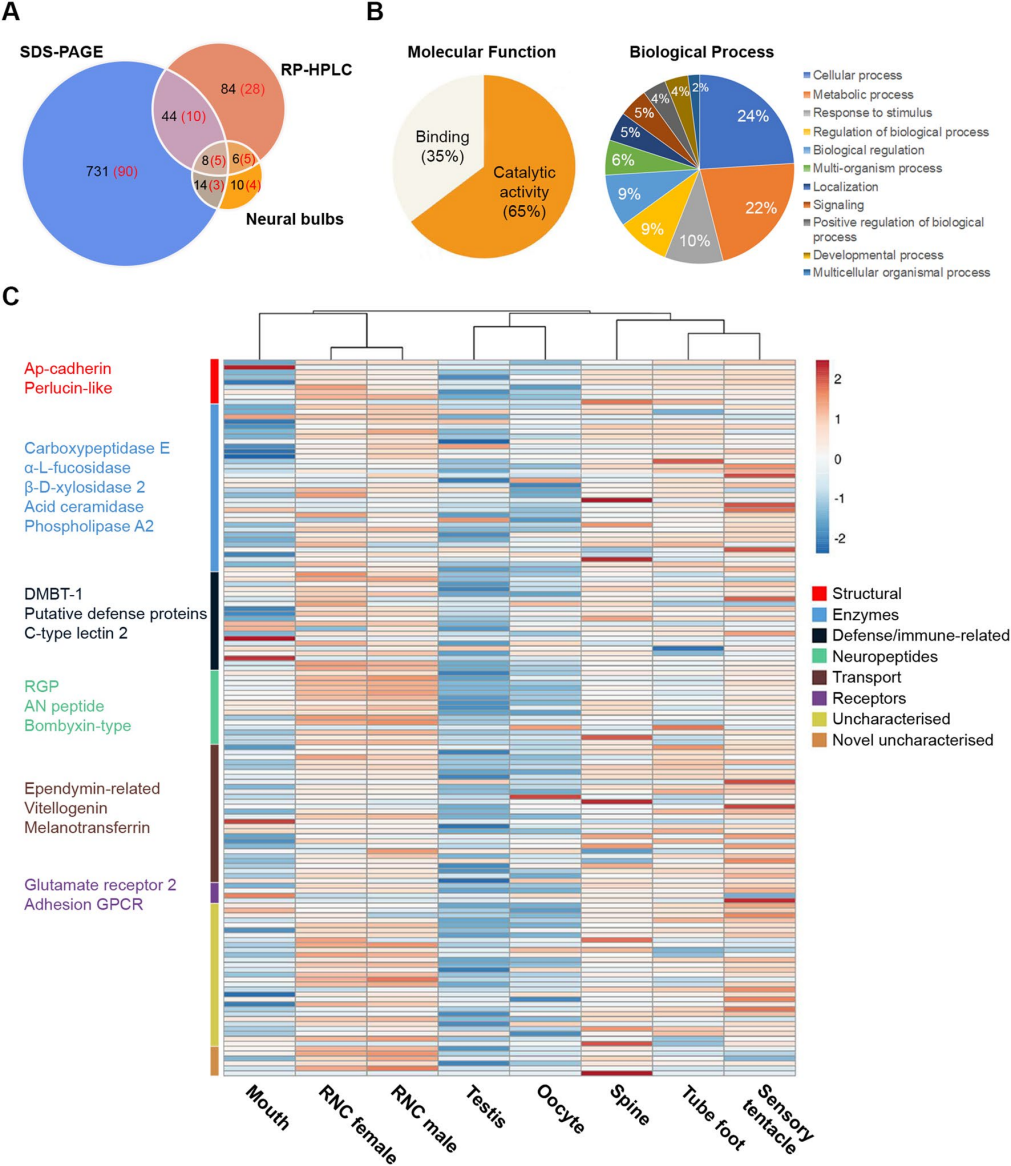
Analysis of putative secreted proteins identified in adult COTS radial nerve cord (RNC). (**A**) An area-proportional Venn diagram showing distribution of total RNC proteins identified from soluble (RP-HPLC) and insoluble (SDS-PAGE) RNC preparations and neural bulbs. In parenthesis is the number of predicted secreted proteins. (**B**) BLAST2GO analysis with presentation of molecular function and biological process. (**C**) Heatmap showing relative tissue gene expression of each identified protein, with scale bar representing the z-score values calculated based on log_2_-transformed expression (Transcripts Per Million) across all tissues. Along the sides of the heatmap, (*right*) groups of genes are categorized based on general functions, and (*left*) gene representatives from each group are shown. Full list of gene names and gene expression are provided in Table S4.

**Figure 6:**
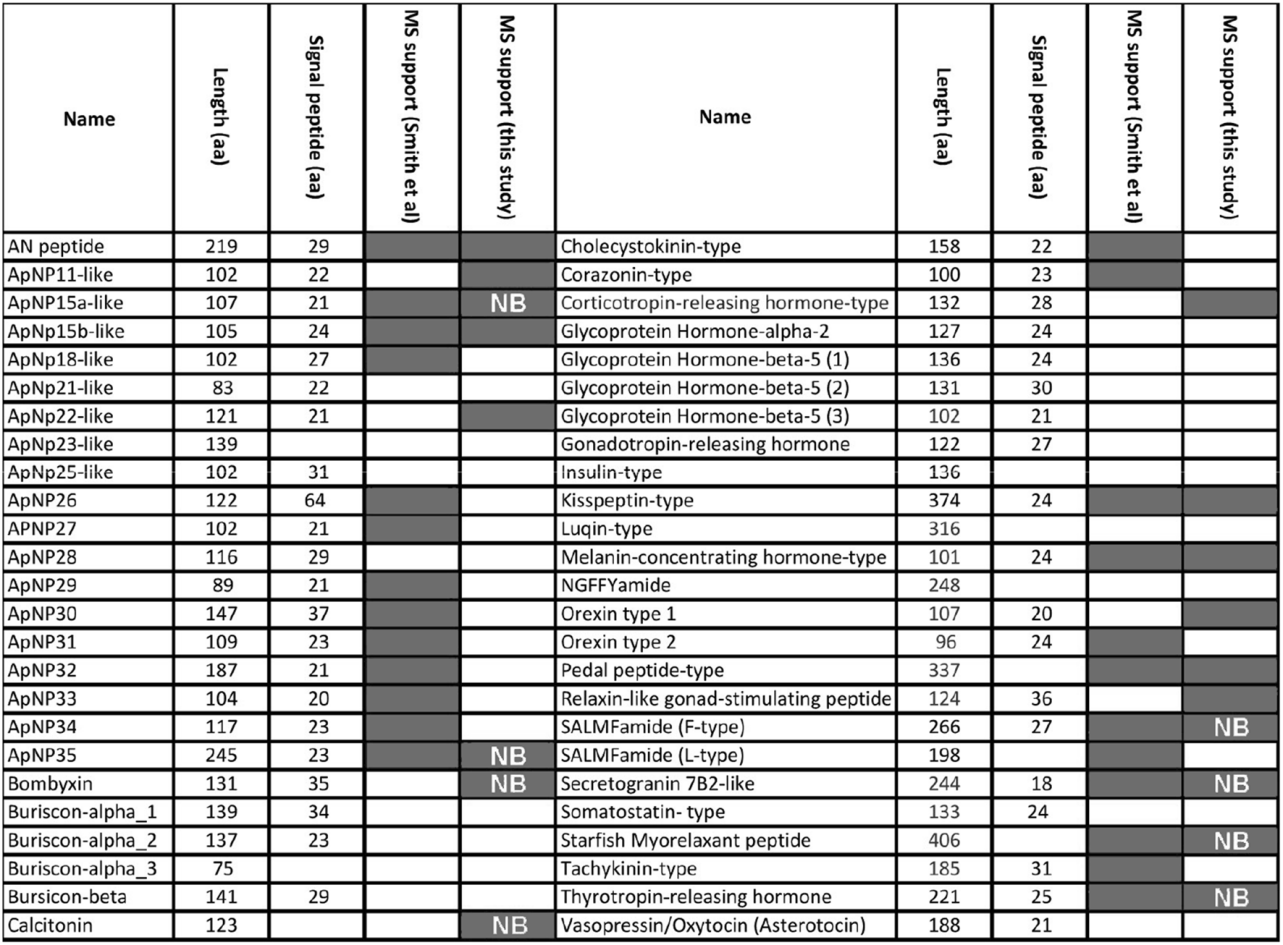
Summary of COTS neuropeptide precursors and those with mass spectral support (grey shading) in adult radial nerve cord. ‘NB’ indicates neuropeptides found in the neural bulbs; ‘aa’, amino acids.

**Figure 7:**
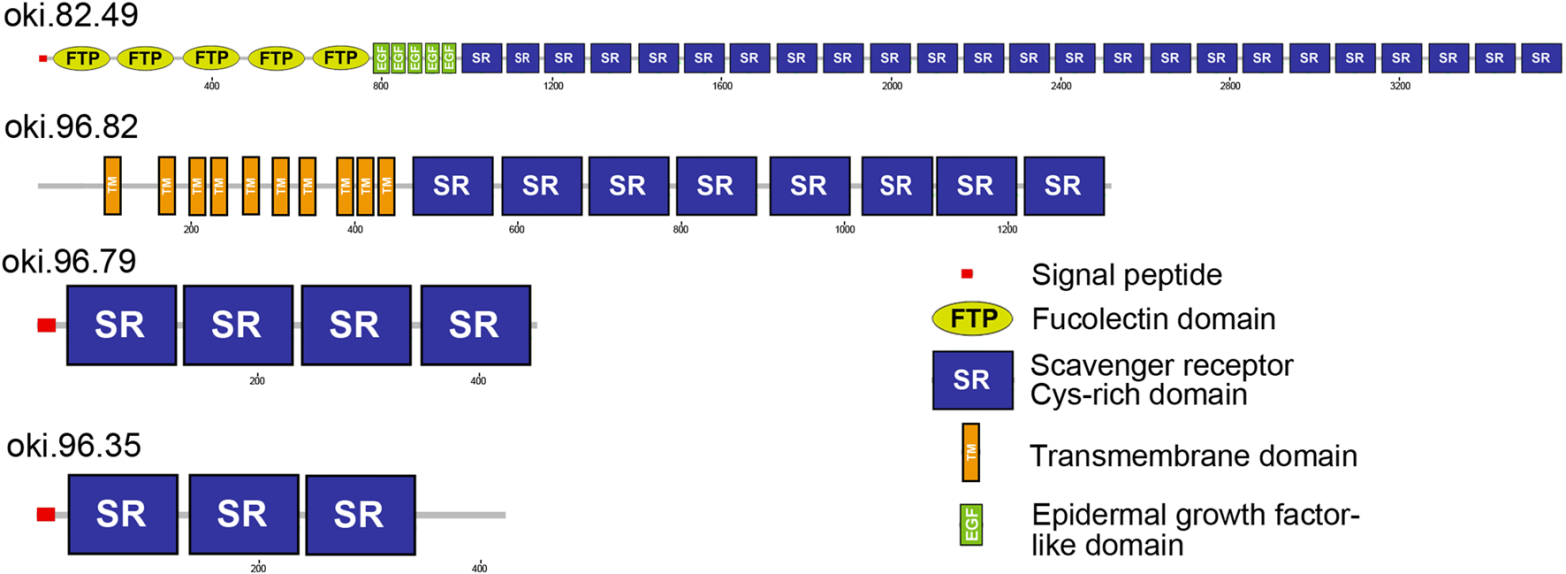
Precursor schematics of deleted in Malignant Brain Tumor 1 (or DMBT1)-like proteins identified from the adult COTS radial nerve cord.

Purification and identification of soluble and insoluble proteins present in the adult COTS RNC, led to the identification of 897 proteins (Fig. 5A and Table S1–S3), of which the majority were identified from the insoluble (SDS-PAGE) RNC preparations. An in silico analysis of these to identify predicted secreted proteins (proteins that contain a signal peptide) revealed 142 putative secreted proteins (Fig. 5A); 34 possessed an annotated molecular function, with 65% being involved in catalytic activity and the remaining 35% in binding (Fig. 5B). Regarding biological processes, 80 proteins were annotated to various processes, including cellular and metabolic processes (24% and 22%, respectively), while 5% of proteins were associated with signalling processes. In general, the predicted secreted proteins fit into functional categories associated with structure, enzymes, defence/immune-related, neuropeptides, transport and uncharacterised or novel uncharacterised. Their gene expression within various adult tissues indicated relatively high expression within the RNC, as well as spine, tube foot, and sensory tentacle tissues (Fig. 5C). Among the neuropeptide precursors annotated, and not previously reported in COTS^18^ were ApNp11, ApNp22-like, bombyxin-type, calcitonin-type, corticotropin-releasing hormone (CRH)-type, orexin type-1, and gonad-stimulating peptide (RGP) (Fig. 6). More specifically, proteins detected in the neural bulbs included several neuropeptide precursors (bombyxin-type, calcitonin-type, TRH-type, F-type SALMFamide, starfish myorelaxant peptide, secretogranin 7B2-like, Ap15a-like, ApNp35) and two Deleted in Malignant Brain Tumor 1 (DMBT1)-like proteins (oki.96.79 and oki.83.49), which belong to the Scavenger Receptor Cys-Rich (SRCR) superfamily^28,29^. An additional DMBT1-like protein was identified in the RNC (oki.96.82) yet did not contain a signal peptide. All DMBT1-like proteins contained Scavenger Receptor (SR) domains (varying from 3 to 24 domains), while the presence of fucolectin domains, transmembrane domains and epidermal growth factor-like domains was variable (Fig. 7).

## Discussion

In this study, we investigated the histology and ultrastructure of the RNC in COTS and confirm the presence of unique bulbous structures in adult COTS. There is no evidence of similar structures in similar studies of the RNCs of other phylogenetically diverse seastars including, *Meridiastra gunnii*^30^, *Echinaster sepositus*^31^, *Echinaster echinophorus*^32^, *Patiria pectinifera*^33^, *Asterias rubens*^34^, *Marthasterias glacialis*^35^, *Patiriella regularis* and *Parvulastra exigua*^36^; to name a few species that all have the smooth epithelial surface, typical of the asteroid RNC. These comparisons provided strong evidence that the neural bulbs of COTS are a novel innovation of this seastar. Meanwhile, neural bulbs were absent from juvenile COTS RNC, suggesting that the neural bulbs were only developed when COTS become an adult. Interestingly, we found no evidence for the presence of the bulbous structures in the closely related adult *A. brevispinus*^37^. The evolutionary divergence between these two *Acanthaster* species has been established by molecular data (approximate similarity of 89% from mitochondrial genomic data)^38,39^, as well as their disparate morphology^37^. In spite of that, they do retain the capacity for interbreeding, which suggests they have undergone recent speciation^37^.

COTS are unusual in comparison to most seastars in that they are specialised corallivores, being able to digest the waxy lipid coral tissue due to a specialised stomach enzyme system^40^. Since the neural bulbs extending form the RNC were absent in both the early juvenile stage of COTS as well as the adult *A. brevispinus*, neither of which feed on coral, we could therefore speculate that the possession of the neural bulbs may be related to diet and habitat. COTS starts benthic life as a herbivore with a preference for coralline algae^41,42^, while *A. brevispinus* feeds on molluscs and detritus^37^. Juvenile COTS transition from herbivory into coral-feeding “sub-adults” at ~ 6 months of age^43^, although this perilous transition can be delayed for years when coral diet is limited^44^. Corals are a hostile, highly defended food source, enveloped in allelochemicals and stinging cnidae, with many species also possessing sweeper tentacles and/or mesenterial filaments^40^. Corals often injure juvenile COTS as they make the dietary switch, including central disc lesions and loss of arms that may lead to death^41^. We hypothesised that the RNC bulbs may be neural adaptations to traversing and feeding on coral. They may function as counter-defences that enable the successful predation and ecosystem co-habitation of COTS with corals. Thus, it is plausible that the contents of the neural bulb cells could have defensive and/or offensive roles, thereby protecting the RNCs from coral-derived neurotoxins and/or releasing neurochemicals that inhibit or suppress the coral defence mechanisms.

Aside from the neural bulbs, the histology and ultrastructure of the RNC of COTS is similar to that of other seastars. The hyponeural region of the RNC for other seastars contains motor neurons^27,34,45^ and for the seastar *A. rubens* it has been shown that these express a variety of neuropeptides^46^. The underlying ectoneural portion of the RNC is by far the largest of the three regions in seastars. In COTS and *A. rubens*^4^*,* its border with the connective tissue layer is quite level, in stark contrast to the vacillating boundary observed in the seastar *P. pectinifera*^47^ and the sea cucumber *Eupentacta fraudatrix*^48^. The fibre bundles have been identified as being an intracellular component of bipolar glial cells^4,49^. Although the presence of glia in echinoderm nervous tissue was repeatedly dismissed by prominent researchers in the field^3,50^, they are suggested to be radial glial-like cells based on immunostaining^4,49^. However, they appear to be different from vertebrate glial cells. Within the ectoneural neuropile was a heavy meshwork of smaller neurons, dendrites, vacuoles, glial fibre bundles and electron dense vesicles. Surrounding the uppermost portion of these fibre bundles were sensory knobs comprising of relatively enlarged nerve fibres that contain an abundance of synaptic vesicles^47^. The non-neuronal bipolar cells within the ectoneural layer are secretory and show immunoreactivity to Reissner’s substance, distinguishing them as a distant relative of the radial glial cells found in chordates^4,49^. The lowermost region of the ectoneural layer is covered by a thick, microvilli-edged neuroepithelium occupying more space than the hyponeural plexus and connective tissue layer combined. The goblet-like cells interspersed within the epithelial portion of this layer potentially function to secrete mucous for microvilli on the epithelial surface. Importantly, the bulbous structures project from the ectoneural tissue and may serve to increase the regions surface area, a common biological trope in structuring tissue.

The neural bulbs appeared to be dominated by secretory cells and vacuoles, the cells with clear vesicles whose contents may have been lost in processing and cells with large vesicles containing protein-like secretory material destined for exocytosis at the surface. The large vesicles may indicate the presence of goblet-like cells which are known to secrete mucus, while the vesicles with dense protein-like inclusions indicated the presence of secretory cells with material in various stages of maturation^51,52^. The juxtaposition of these cells is reminiscent of the duo-glandular system seen in seastar tube feet^53^. Cells within the epithelial portion of the neural bulbs were known to produce neurotransmitters such as serotonin and GABA (gamma-aminobutyric acid)^19^.

Our proteomic analysis of the adult COTS RNC, including the neural bulbs, identified almost 900 proteins, 16% of which were predicted to be secreted, with a significant number known to be involved in cellular and metabolic processes, including structural, enzymatic and defence/immune-related. Tissue gene expression indicated that besides RNC, the tube feet, sensory tentacles and spine had relatively high gene expression levels. The COTS spine is well recognised for its role in predator physical defense^54^, however, a role in sensory detection and neural transmission should be explored. Regarding ependymin-related proteins (EPDRs), COTS are known to have expanded significantly through tandem duplication^55^, and due to their presence in COTS-conditioned seawater, it was hypothesised that they could play a role in conspecific communication^22^. Several of these COTS EPDRs were also found in our RNC proteome, suggesting that the RNC could be a potential source. In the vertebrates, EPDRs appear to function in hydrophobic molecule binding, based on structural and in silico interaction analysis^56^. A previous study by Smith et al.^18^ analysed neuropeptides in the adult COTS RNC based on a molecular weight cut-off prior to MS analysis. Our RP-HPLC and LC–MS/MS workflow yielded similarities to some of these neuropeptides, although some additional neuropeptides were also found, including ApNp11, ApNp22-like, bombyxin-type, calcitonin-type, CRH-type, orexin type-1, and RGP.

Providing a lower signal-to-biological-noise ratio caused by sample complexity, the more targeted analysis of isolated neural bulbs allowed for the identification of proteins that were specific or relatively abundant in the neural bulbs. These included several neuropeptides such as ApNP15a, calcitonin-type, F-type SALMFamide, myorelaxant peptide, TRH-type, and bombyxin. The latter was exclusively detected in neural bulbs, suggesting that the neural bulbs may be a major production and/or secretion site of this neuropeptide. Interestingly, a few neuropeptides detected in the neural bulbs were functionally annotated as myorelaxant agents (e.g., myorelaxant peptide, SALMFamide, and calcitonin-type peptides). A myorelaxant peptide has been found in the RNC and neuromuscular tissues of the *P. pectinifera,* where it stimulates muscle relaxation^57^. Similarly, SALMFamide peptides, which were the first echinoderm neuropeptides to be fully sequenced^58^ and are derived from L-type (LxFamide motif) or F-type (FxFamide motif) precursor proteins, also act as myorelaxants^59^. Calcitonin is traditionally known for its role in vertebrate calcium metabolism^60^ and calcitonin-type peptides have been identified in several echinoderm species^61–63^. Furthermore, it was recently discovered that calcitonin-type neuropeptides act as muscle relaxants in the seastars *A. rubens* and *P. pectinifera*^34^. Therefore, we hypothesised that the neural bulbs might associate with a regulation of muscle function in COTS.

Four DMBT1-like proteins were elucidated from the adult COTS RNC, two of which were present in neural bulb preparations. Although DMBT1-like proteins have not been adequately investigated in echinoderms, it is known from other eumetazoans that the *Dmbt* gene encodes alternatively spliced glycoproteins associated with the membrane or products of epithelial secretions^64^. These glycoproteins have been individually identified in both terrestrial and aquatic animal species [named DMBT1, salivary agglutinin (SAG), crp-ductin, gp-340, ebnerin, vomeroglandin, hensin, and muclin], where they have a variety of functions^64^. *Dmbt1* tissue expression levels are highly species and organ dependent^64^. For example, in mice, the *Dmbt1* (known as vomeroglandin) is actively expressed within the vomeronasal glands, important for pheromone perception among vertebrates^65,66^. In humans, SAGs aggregate and bind to a large variety of microorganisms such as viruses, bacteria and fungi^67^. In adult COTS*,* it is possible that DMBT1-like proteins provide defence against foreign microbes or upon exposure to coral. The COTS genome contains a total of 56 genes that encode proteins annotated as DMBT-like, and 124 proteins that contain one or more SRCR domains^22^. By comparison, humans contain 22 SRCR-containing proteins, while the purple sea urchin *S. purpuratus* has 407^22^. This suggested an expansion of DMBT/DMBT-like proteins in the echinoderm lineage.

## Conclusions and future directions

We have obtained new anatomical and proteomic insight into the COTS RNC. Significantly, we have demonstrated that the adult COTS RNC oral surface is overlaid with neuroepithelial bulbs that are protrusions of the ectoneural tissue. Furthermore, the neuroproteomic data from adult COTS RNC affords a helpful resource to address the functional protein (including neuropeptide) networks and molecular mechanisms that underlie physiological, anatomical, and behavioural processes. For instance, nerve tissues are known to play important roles in regeneration in both vertebrates and invertebrates, providing a phenomenon called nerve-dependent regeneration^68,69^. Those proteins identified in the adult neural bulb, such as neuropeptides and DMBT1-like proteins, are excellent initial candidates for further exploration. Although this study further expanded our understanding of the basic neurobiology of COTS, continued research into their morphological, as well as behavioural, physiological and ecological traits, is essential for improving control methods.

## Supplementary Information

Supplementary Information 1.

Supplementary Information 2.

## Data Availability

All data generated or analysed during this study are included in this published article (and its Supplementary Information files). Mass spectrometry proteomics raw data obtained from this study are available on the PRoteomics IDEntifications (PRIDE) Archive database PXD005837.
