# Author Correction: Structure and proteomic analysis of the crown-of-thorns starfish (*Acanthaster sp.*) radial nerve cord

**DOI:** 10.1038/s41598-023-34881-7

**Published:** 2023-05-11

**Authors:** Meaghan K. Smith, Bronwyn A. Rotgans, Tomas Lang, Ryan Johnston, Tianfang Wang, Saowaros Suwansa-ard, Utpal Bose, Nori Satoh, Michaela Egertova, Michael R. Hall, Maria Byrne, Maurice R. Elphick, Cherie A. Motti, Scott F. Cummins

**Affiliations:** 1 Centre for Bioinnovation, University of the Sunshine Coast, Maroochydore DC, QLD 4558 Australia; 2 School of Science, Technology and Engineering, University of the Sunshine Coast, Maroochydore DC, QLD 4558 Australia; 3 Okinawa Institute of Science and Technology, Okinawa, Japan; 4 School of Biological and Chemical Sciences, Queen Mary University of London, London, UK; 5 Australian Institute of Marine Science (AIMS), Cape Ferguson, Townsville, QLD 4810 Australia; 6 School of Life and Environmental Sciences, University of Sydney, Sydney, NSW 2006 Australia

Correction to: *Scientific Reports* 10.1038/s41598-023-30425-1, published online 27 February 2023

The original version of this Article contained an error in the spelling of the author Maria Byrne which was incorrectly given as Maria Bryne.

The original Article has been corrected.

