# Supplementary figures and images for: Structure and proteomic analysis of the crown-of-thorns starfish (Acanthaster sp.) radial nerve cord
Source: Sci Rep. 2023 Feb 27;13:3349. doi: 10.1038/s41598-023-30425-1 (PMC9971248; doi:10.1038/s41598-023-30425-1)

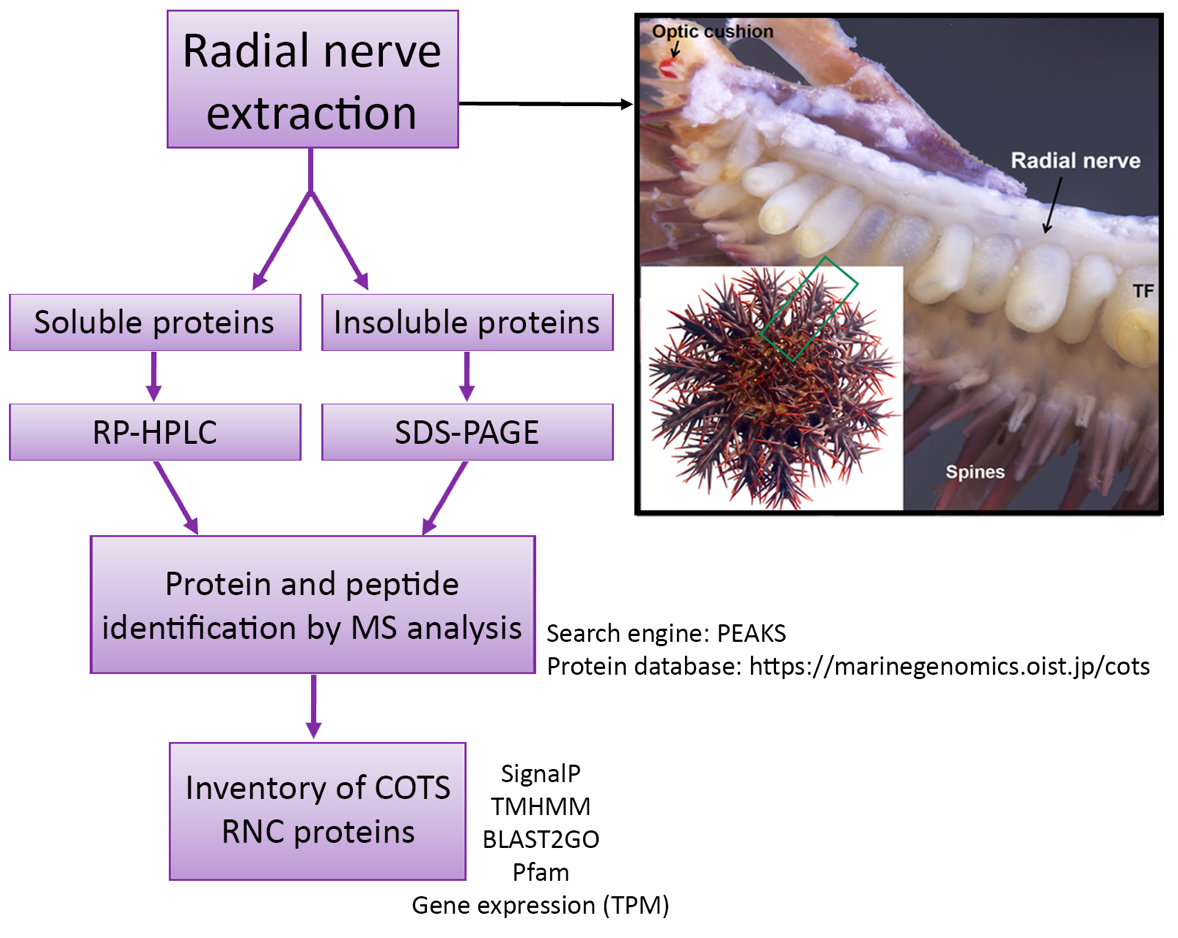


**Figure S1.** Overall workflow for the proteomic analysis of the adult *A. cf. solaris* RNC.

Supplement: Supplementary file 1 — Supplementary Information 1. [file 41598_2023_30425_MOESM1_ESM.docx]
